# Themis differentially regulates T follicular helper cell differentiation during early and late stages of chronic viral infection

**DOI:** 10.3389/fimmu.2025.1638178

**Published:** 2025-07-24

**Authors:** Yuzhen Zhu, Yuzhou Bao, Ning Wang, Qifeng Gan, Jian Tang, Yu Cong, Bowen Hou, Minxue Quan, Chaonan Yan, Siyi Liu, Shuo Lin, Xiaobin Zhang, Yanping Du, Lichao Hou, Nicholas R. J. Gascoigne, Bing Xu, Guo Fu, Qifan Zheng

**Affiliations:** ^1^ Department of Hematology, The First Affiliated Hospital and Institute of Hematology, School of Medicine, Xiamen University, Xiamen, China; ^2^ Department of Pulmonary and Critical Care Medicine, Zhongshan Hospital of Xiamen University, School of Medicine, Xiamen University, Xiamen, Fujian, China; ^3^ State Key Laboratory of Cellular Stress Biology, School of Medicine, Faculty of Medicine and Life Sciences, Xiamen University, Xiamen, China; ^4^ Immunology Center of Georgia (IMMCG), Augusta University, Augusta, GA, United States; ^5^ Xiang’an Hospital of Xiamen University, Xiamen University, Xiamen, China; ^6^ Immunology Translational Research Programme and Department of Microbiology and Immunology, Yong Loo Lin School of Medicine, National University of Singapore, Singapore, Singapore; ^7^ Cancer Research Center, School of Medicine, Xiamen University, Xiamen, China

**Keywords:** THEMIS, CD4 + T cell progenitor, Tfh cells, chronic viral infection, GC responses

## Abstract

**Introduction:**

T follicular helper (TFH) cells are critical for humoral immunity during chronic viral infection, but the mechanisms guiding their differentiation from a novel CD4⁺ T cell progenitors remain incompletely understood. Themis, a T cell-specific adaptor protein, has been implicated in T cell development and function, but its role in peripheral CD4⁺ T cell differentiation under chronic antigen stimulation has not been defined.

**Methods:**

We used a chronic lymphocytic choriomeningitis virus (LCMV) Clone13 infection model in wild-type and Themis conditional knockout (cKO) mice. A combination of adoptive cell transfer, flow cytometry, histological analysis, and single-cell RNA sequencing (scRNA-seq) was applied to analyze the differentiation of CD4⁺ T cells into TFH cells at multiple infection stages.

**Results:**

Themis expression is strongly upregulated in TFH cells at early stages of infection, and as expected, Themis promotes TFH cell differentiation at this stage. However, unexpectedly, at the late stages of chronic LCMV infection, Themis-deficient CD4+ T cells favored TFH cell differentiation and helped control the virus by enhancing GC responses and antibody production, suggesting that Themis inhibits TFH cell differentiation at this stage. In the late stage we found that Themis inhibits the differentiation of CD4+ T cell progenitors into TFH cells through transcriptional regulation.

**Discussion:**

Our study uncovers a dual-stage regulatory role of Themis in TFH cell differentiation during chronic viral infection. While promoting TFH generation early, Themis unexpectedly restrains excessive differentiation at later stages, suggesting its function is context- and time-dependent. These findings highlight Themis as a key temporal regulator of CD4⁺ T cell fate decisions under chronic antigenic stress.

## Introduction

CD4^+^ T cells provide critical help in most, but not all, immune responses (1–3). One of the best examples reflecting the different requirements for CD4^+^ T cell help is LCMV infection of mice. For example, in acute LCMV infection, CD8^+^ T cells alone generate a powerful antiviral immune response without the help of CD4^+^ T cells and can essentially contain the virus (4–6). In contrast, in chronic LCMV infection, as CD8^+^ T cells gradually become dysfunctional, CD4^+^ T cells are required to help B cells produce neutralizing antibodies, and ultimately the three cell types work together to eliminate the persistent virus (7, 8). The dysfunctional state of CD8^+^ T cells, termed exhaustion, is common in chronic infections and cancer (9, 10) and is exacerbated if CD4^+^ T cells are depleted prior to infection (11–13), but can be alleviated by replenishing with fresh CD4^+^ T cells (14). Similarly, lack of CD4^+^ T cell help contributes to CD8^+^ T cell dysfunction in human chronic viral infections (15, 16). Although these studies highlighted the importance of CD4^+^ T cells in CD8^+^ T cell exhaustion, their own exhaustion is relatively poorly understood (17–20). This is partly because CD4^+^ T cell exhaustion is more complex.

One complicating factor in CD4^+^ T cell exhaustion is its heterogeneity, as some CD4^+^ T cells become functionally inactivated early in infection, whereas others remain functional throughout infection (18, 21). Another complicating factor is that CD4^+^ T cell exhaustion manifests as loss of some functions (such as IL-2, TNF, and IFNγ production) (17, 18) but gain or maintenance of other functions (such as IL-10 and IL-21 production) (22–25). In accordance with their complex phenotype, exhausted CD4^+^ T cells also have a unique transcriptional profile that differs not only from effector and memory CD4^+^ T cells but also from exhausted CD8^+^ T cells (20). These further suggest that the underlying mechanisms of CD4^+^ T cells and CD8^+^ T cells exhaustion may be different. In fact, studies have shown that during persistent viral infection, CD4^+^ T cells do not become typical exhausted cells, but instead gradually change their differentiation. That is, these CD4^+^ T cells redirect their differentiation from an initial T_H_1 phenotype to a T follicular helper (T_FH_) cell phenotype, an important shift that is thought to promote antibody production and resolve viremia without provoking T cell-mediated immunopathology (26–30). This concept is further substantiated by recent studies showing that IL-21 produced by T_FH_ cells, rather than T_H_1 cells, plays a critical role in maintaining CD8^+^ T cell functionality during chronic viral infections (31). Another important advance in the study of CD4^+^ T cell exhaustion is the recent discovery of a self-renewing progenitor population (Tprog) that can sustain the differentiation of T_FH_ cells and non-T_FH_ effector CD4^+^ T cells (31, 32). However, the details and players of this newly proposed differentiation pathway remain unclear. In contrast, a similar population of progenitors of exhausted cells (T-pex) was clearly identified and has been extensively studied in CD8^+^ T cells (9, 10).

Themis is a T cell-specific adaptor protein that plays important roles in thymocyte development (33–36). In mature CD8^+^ T cells, Themis is required to maintain their homeostasis (37), responsiveness to TCR stimulation (38, 39) and to common γ-chain cytokines (40). More strikingly, in a chronic LCMV infection model, we recently found that CD8^+^ T cells lacking Themis perturbed their own exhaustion, leading to 80% mouse mortality (41). This is because Themis deficiency causes CD8^+^ T cells to differentiate into effector cells with enhanced function, while the formation of CD8^+^ T-pex cells is impaired. Interestingly, in this model, we also noted that depletion of CD4^+^ T cells reduced the mortality of infected Themis T-cell conditional knockout mice by 20% (41), suggesting that Themis-deficient CD4^+^ cells may play some role in chronic LCMV infection. In this study, we systematically investigate whether and how Themis affects CD4^+^ T cells function during chronic LCMV infection, and report a novel role for Themis in differentially regulating T_FH_ cell differentiation during early and late stages of chronic viral infection.

## Materials and methods

### Mice

Themis^flox/flox^ dLck-cre mice (cKO) were generated and kindly provided by Dr. Nicholas Gascoigne at the National University of Singapore. C57BL/6J (#000664), *Rag1* ^–/–^ mice (#034159), CD45.1 (#002014), SMARTA mice (#030450) were purchased from the Jackson Laboratory and maintained in Xiamen University Laboratory Animal Center. All mice were bred and maintained under specific pathogen-free conditions at the animal center at Xiamen University. All experiments were performed using sex-matched littermates ages between 6 and 12 weeks and were approved by the Institutional Animal Care and Use Committee of Xiamen University.

### Antibodies and regents

All antibodies used for flow cytometry staining were purchased from BioLegend, eBioscience, Miltenyi Biotec and BD Biosciences: anti-CD16/CD32 (2.4G2), anti-CD4 (RM4-4), anti-CD8 (53-6.7), anti-CD45.1 (A20), anti-CD45.2 (104), anti-CD44 (IM7), anti-CD62L (MEL-14), anti-PD-1 (29F.1A12), anti-CX3CR1 (SA011F11), anti-TCF-7/TCF-1 (S33-966), anti-CD101 (Moushi101), anti-CD19 (D3), anti-B220 (RA3-6B2), anti-CD38 (90), anti-IgM (RMM-1), anti-IgD (IA6-2), anti-CD95 (Jo2), anti-CD138 (281-2), anti-GL7 (GL7), anti-CXCR5(2G8), rat IgG2a (MRG2a-83), anti-SLAM(TC15-12F12.2), anti-ICOS(15F9), anti-Bcl-6(K112-91), biotinylated anti-rat IgG (553894), APC Streptavidin (405207), Annexin V/7-AAD kit (559763), CFSE (C34554), CTV (C34557). The viral peptide gp33-41 (KAVYNFATM) and gp61-80 (GLKGPDIYKGVYQFKSVEFD) were purchased from GenScript. LCMV-specific MHC-I tetramers H-2D^b^/gp33–41 were purchased from Reagent platform of Cancer Research Center of Xiamen University. PMA (phorbol 12-myristate 13-acetate) was purchased from Sigma. Ionomycin was purchased from Yeasen.

### Viral infection and titers

LCMV Armstrong (LCMV Arm) and LCMV Clone13 (LCMV C13) strains were propagated in BHK-21 cells and titrated on Vero cells. For infection, 2×10^5^ plaque-forming units (PFUs) of LCMV Arm were intraperitoneally injected into mice to establish an acute viral infection, 2×10^6^ plaque-forming units (PFUs) of LCMV C13 were intravenously injected into mice to establish a chronic infection in mice. In adoptive transfer experiments, recipient mice were infected 1 day after cell transfer.

Viral titers were determined in mouse serum by qPCR assay as previously described (42). Briefly, a standard curve was generated by serial dilution of a virus stock with a predetermined titer. RNA was extracted from standard samples and tested samples using a TIANamp virus RNA kit (TIANGEN, no. DP315-R), cDNA was synthesized using the primer gp33 (reverse; 59-CATTCACCTGGACTTTGTCAGACTC-39) and TransScript first-strand cDNA synthesis supermix (TransGen Biotech, AT301-03). Virus titers were detected by quantitative PCR with the primers gp33 (forward; 59-GCAACTGCTGTGTTCCCGAAAC-39) and gp33 (reverse) and then converted to PFU/ml according to the standard curve.

### Naïve CD4^+^ T cell purification

Naïve CD4^+^ T cells were purified using a negative selection strategy. Briefly, single-cell suspensions were freshly isolated from spleens and lymph nodes, followed by red blood cell lysis. A panel of anti-mouse biotinylated antibodies was used to deplete non-CD4^+^ T cells, including anti-B220 (RA3-6b2), anti-CD8 (53-6.7), anti-CD11b (M1-70), anti-CD11c (N418), anti-CD25 (PC61.5), anti-CD44 (IM7), anti-NK1.1 (PK136), anti-Ly-6G/Ly-6C (RB6-8C5), anti-F4/80 (BM8), and anti-TER119 (Ter119). All antibodies were sourced from BioLegend. The cells were incubated with the antibodies for 20 minutes at 4°C, followed by a 10-minute incubation with streptavidin-conjugated magnetic beads (BeaverBeads Streptavidin, Beaver, 22307-10). The cell-bead mixture was placed on a magnetic stand (DynaMag™-2, ThermoFisher, 12321D), allowing unbound cells to be aspirated off, while naïve CD4^+^ T cells were retained and subsequently collected. Purified naïve T cells were phenotypically confirmed as CD4^+^CD62L^hi^ CD44^lo^ by flow cytometry, achieving a purity exceeding 95%.

### CFSE labeling of naïve SMtg CD4^+^ T cells

Freshly isolated naïve SMtg CD4^+^ T cells were labeled with CFSE according to the manufacturer’s protocol. Briefly, cells were washed once in prewarmed phosphate-buffered saline (PBS, 37°C) and centrifuged at room temperature to collect the pellet. The cell suspension was adjusted to a concentration of 2–3 × 10^6^ cells/ml in prewarmed PBS. CFSE dye was then added, and the cells were incubated in a 37°C water bath for 10 minutes. Following labeling, excess dye was removed by washing the cells with PBS and culture medium. The labeled cells were resuspended and prepared for downstream experiments.

### Lymphopenia-induced proliferation assay

Naïve SMtg WT and cKO cells, marked with distinct congenic labels, were mixed at a 1:1 ratio, labeled with CTV, and adoptively transferred into preconditioned recipient mice via intravenous injection. Recipient mice, preconditioned by irradiation with 5.5 grays using RS2000 160-keV equipment (Rad Source Technologies), were *Rag1* ^–/–^ hosts. At designated time points post-transfer, donor cells from recipient mice were analyzed by flow cytometry.

### Histology and sectioning

Spleens from infected mice were harvested and fixed in PBS-buffered formalin before embedding in paraffin. 5 μm tissue sections were prepared and stained with H&E following the manufacturer’s protocol (Boster, AR1180-100). Microscopic images were captured using a Motic VM1 digital slice scanning microscope.

### Immunofluorescence staining

After treatments as indicated in the figure legends, spleens were fixed in BD Fix/Perm Solution (Cat No: 51-2090KZ) at 4°C overnight. Fixed spleens were washed with PBS for 10 minutes on a shaker, followed by incubation in 30% sucrose (Sigma, V900116) overnight at 4°C. The tissues were embedded in OCT (Sakura, 4583), sectioned at a thickness of 7 μm using a Leica CM1950 cryostat, and permeabilized with 0.3% TritonX-100 in PBS for 10 minutes. Blocking was performed with 5% BSA in PBS for 60 minutes at room temperature. Sections were immunostained with antibodies targeting IgD, CD3, and GL7, incubated in a dark chamber for 24 hours at room temperature. Confocal imaging was conducted using a Leica TCS SP8 microscope.

### Flow cytometry analysis and sorting

Single-cell suspensions were prepared from spleens, thymuses, and peripheral lymph nodes, followed by red blood cell lysis. Cells were resuspended in RPMI 1640 medium containing 2% FBS. For surface marker staining, antibodies were added to cells and incubated for 30 minutes at 4°C, followed by washing with flow cytometry staining buffer. CXCR5 staining was performed in three steps: incubation with purified anti-CXCR5, followed by biotinylated anti-rat IgG, and finally APC-labeled streptavidin. Each step was performed at 4°C in CXCR5 staining buffer (PBS with 0.5% BSA, 2% FCS, and 2% normal mouse serum). Intracellular transcription factor staining was conducted after surface marker staining by fixing and permeabilizing cells in Fix/Perm buffer (eBioscience, 00-5521-00) for 30 minutes, followed by incubation with transcription factor antibodies for 1 hour at room temperature.

SMtg WT and cKO cells (0.5 × 10^4^ of each type) were intravenously injected into B6 hosts, which were infected with LCMV C13–24 hours later. At 21 dpi, SMtg WT and cKO cells were sorted from 15 mice. For sorting, CD4^+^ T cells were negatively selected and stained with fluorochrome-conjugated antibodies in sorting buffer (PBS with 1 mM EDTA, 25 mM HEPES, and 1% FBS). Sorting was performed using a BD FACSAria Fusion sorter (BD Biosciences). Flow cytometry data were acquired on a Fortessa or LSRFortessa X-20 (BD Biosciences) and analyzed with FlowJo software (version 10, Treestar).

### Enzyme-linked immunosorbent assay

Serum samples were collected at 8, 21, 30, and 40 dpi. LCMV-specific IgG concentrations were determined using ELISA. Microtiter plates (ThermoFisher, 439454) were coated with lysates from LCMV Cl13-infected baby hamster kidney (BHK) cells. Plates were blocked with 3% BSA in PBS, and serum samples were incubated for 2 hours at room temperature. After incubation with biotin-conjugated anti-IgG (Southern Biotech, 1030-08), plates were treated with horseradish peroxidase-conjugated streptavidin (Southern Biotech, 7105-05) for 1 hour, followed by color development using tetramethylbenzidine substrate (Bioleged, 421101). Absorbance was quantified using a SpectraMax Absorbance Reader (BAOCHENG).

### Single-cell RNA-seq library preparation

Single-cell RNA sequencing libraries were prepared using the DNBeLAB V2 kit (BGI Genomics), strictly following the manufacturer’s guidelines (43). Briefly, cells were sorted via FACS and washed once with PBS supplemented with 0.04% BSA, then resuspended in PBS containing 0.04% BSA. Following reverse transcription and cell barcoding in droplets, emulsions were broken, and cDNA was purified. PCR amplification was subsequently performed as follows: 98°C for 45 seconds, followed by 14 cycles of 98°C for 20 seconds, 67°C for 30 seconds, and 72°C for 1 minute, with a final extension at 72°C for 1 minute. The resulting amplified cDNA was then utilized for constructing the gene expression library. Quantification of the scRNA-seq libraries was carried out using the Qubit dsDNA HS Assay Kit (Invitrogen) and a High-Sensitivity DNA chip run on a Bioanalyzer 2100 system (Agilent). Sequencing was performed on the DNBSEQ C4 platform (BGI).

### scRNA-seq library processing

The raw sequencing data (GSE284735) were processed using Cell Ranger (10X Genomics) to generate a gene expression matrix containing UMIs (unique molecular identifiers), following the default parameters. The gene expression UMI matrix was further analyzed in Seurat (version 5.1.0) (44). Cells with a number of captured genes falling within two standard deviations of the mean were retained. Cells with mitochondrial RNA content above the 95 percentile were excluded from further analysis to minimize technical biases.

### Trajectory inference

Trajectory analysis of the CD4+ T cell response during LCMV-c13 infection was conducted using Slingshot2 (version 2.12) in R (45). UMAP-based dimensionality reduction was performed on cells from the 21dpi time point (2 samples) after removing cells from infrequent populations. Slingshot2 was then employed to identify the lineage structure and infer pseudotime for the cells, using the naive phenotype cluster as the root node.

Slingshot2 fits principal curves through the data, enabling the identification of differentiation trajectories based on gene expression profiles. These trajectories were visualized using UMAP to illustrate the progression of CD4+ T cell differentiation across time points (21 dpi). Pseudotime was calculated along the inferred curves, mapping the differentiation process in the context of LCMV-c13 infection. This analysis considered dynamic gene expression changes across different stages of differentiation, with pseudotime values used to order the cells along these trajectories.

### Statistical analyses

All summarized data are shown in graphs with mean ± SEMs. Unpaired or paired Student t tests and ANOVA analyses were used as indicated and GraphPad Prism software (version 8.0). Differences with p-values>0.05 were considered non-significant (ns). p-values<0.05 were considered significant (*p<0.05; **p<0.01; ***p<0.001; ****p<0.0001).

## Results

### Themis expression correlates with T_FH_ cell differentiation

**Figure 1:**
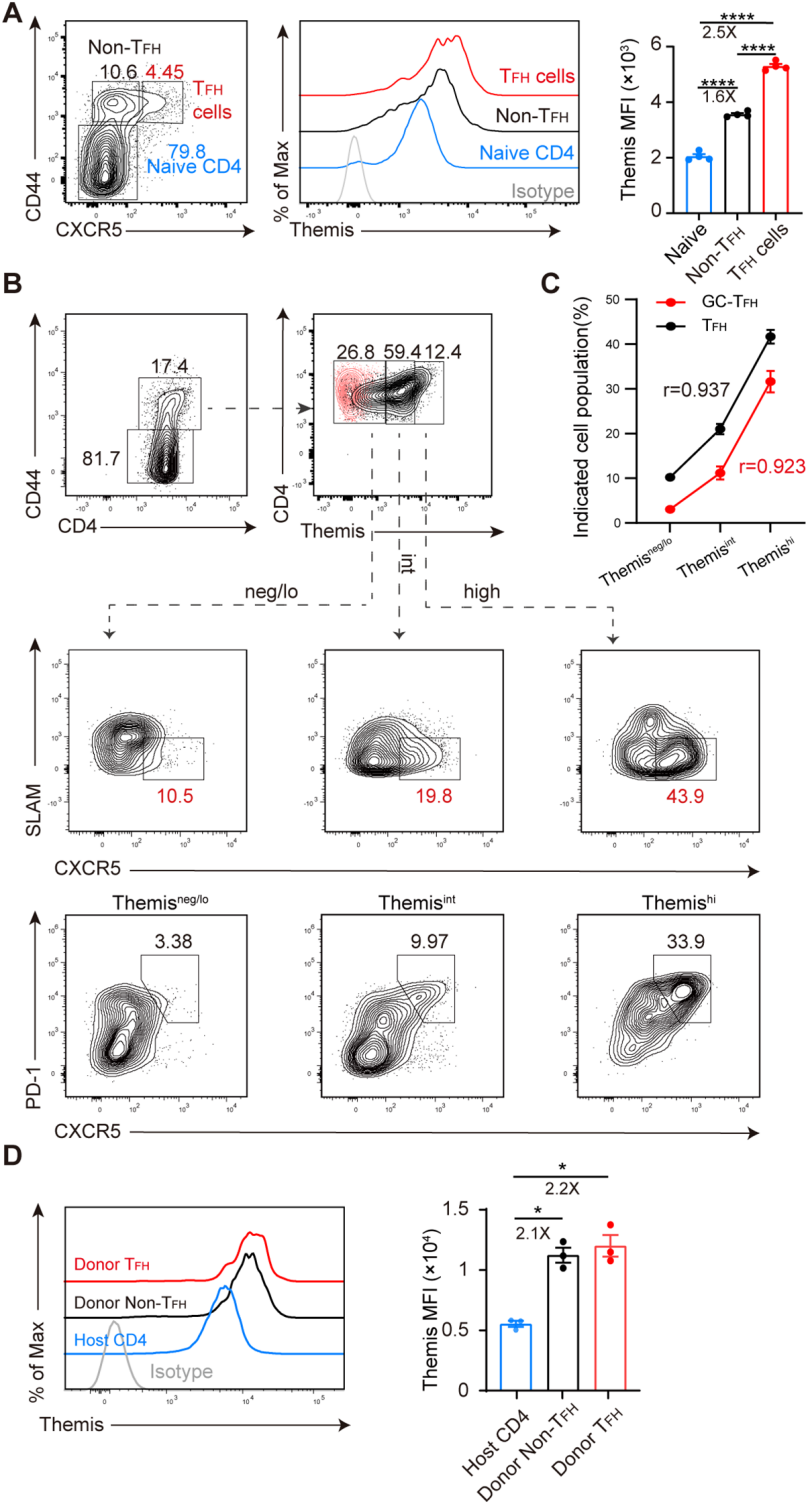
Themis expression is associated with T_FH_ cell differentiation. B6 mice were infected with LCMV C13 and splenic CD4^+^ T cells were analyzed at 5 dpi. **(A)** Shown are representative FACS plots of cell subsets gating (left) and Themis expression in the gated cell subsets (middle) and summary of the mean fluorescence intensity (MFI) of Themis (right). Themis isotype control antibody staining was shown as gray histogram. **(B)** Shown are representative FACS plots of cells gated in Themis^neg/lo^, Themis^int^, and Themis^hi^ compartments (top) and analyzed for CXCR5 and SLAM expression (middle) or CXCR5 and PD-1 expression (bottom). Themis isotype control antibody staining was incorporated and overlaid as red dots. **(C)** Correlation analysis using Pearson’s correlation coefficient (r). Positive r value indicates a direct correlation and negative r value indicates an inverse relationship. **(D)** Themis expression is evaluated in transferred cells. 2×10^6^ naïve SMARTA cells (CD45.1^+^CD45.2^+^) were adoptively transferred into CD45.1^+^ B6 recipients, followed by LCMV C13 infection within 24 hours. Shown are representative FACS histograms of Themis expression in the indicated cell subsets (left) and summary of the MFI of Themis in each cell subsets (right). Data are representative of at least two independent experiments (n=3–5 mice per group). In data summary plots, each symbol represents an individual mouse, the error bars represent the standard errors of the means (SEMs). P value was calculated by one-way ANOVA, *P < 0.05, ****P < 0.0001.

To determine whether Themis is involved in CD4^+^ T cell differentiation in chronic LCMV infection, we evaluated the expression of Themis in different CD4^+^ T cell subsets derived from LCMV C13-infected C57BL/6J (B6) mice. In this infection model, CD4^+^ T cells exhibited a differentiation bias toward T_FH_ cells, which participate in the formation of germinal centers (GC) and promote B cell somatic hypermutation and antibody production (26, 27, 29). We found that at 5 days post infection (dpi), there was a clear effector CD4^+^ T cell (CD44^+^) population emerging while the majority of CD4^+^ T cells were still of naive phenotype (CD44^–^) (**Figure 1A**, left). The effector CD4^+^ T cells were further subdivided into T_FH_ (CXCR5^+^) and non-T_FH_ (CXCR5^–^) cells based on their CXCR5 expression (**Figure 1A**, left). Under this gating strategy, we evaluated the expression of Themis (**Figure 1A**, middle) and found that Themis expression in T_FH_ and non-T_FH_ cells was 2.5-fold and 1.6-fold higher than that in naive CD4^+^ T cells, respectively (**Figure 1A**, right). To obtain more clues, we also divided effector CD4^+^ T cells into Themis^neg/lo^, Themis^int^ and Themis^hi^ subsets (**Figure 1B**, top row), and examined their CXCR5 and SLAM expression (**Figure 1B**, middle row). CXCR5 and SLAM counterstaining more accurately distinguish T_FH_ cells (CXCR5^+^SLAM^–^) (46). This time we found a more evident relationship between Themis expression and T_FH_ cell differentiation, with the Themis^neg/lo^, Themis^int^ and Themis^hi^ subsets having the smallest, intermediate and largest proportions of T_FH_ cells, respectively (**Figure 1B**, middle row). Similar results were obtained when we analyzed GC-T_FH_ (CXCR5^+^PD-1^+^) cells by CXCR5 and PD-1 counterstaining (**Figure 1B**, bottom row). Thus, a strong positive correlation was established between Themis expression and T_FH_ cell differentiation (**Figure 1C**).

Although the above results suggested that Themis may be involved in T_FH_ cell formation, one caveat is that we analyzed bulk CD4^+^ T cells, not virus-specific CD4^+^ T cells. However, due to the extremely low precursor frequency of endogenous virus-specific CD4^+^ T cells (47), we were unable to obtain consistent results by specific MHC-II tetramer staining at 2–3 dpi, when these cells reportedly initiate T_FH_ cell differentiation (48–51). To circumvent this issue, we adopted another approach, transferring SMARTA TCR transgenic cells into B6 mice, followed by LCMV C13 infection. SMARTA cells are CD4^+^, MHC-II-restricted, TCR transgenic cells specifically recognizing LCMV glycoprotein epitope gp61–80 presented by H2-IA^b^ (52). We found that at 3 dpi, Themis expression in T_FH_ (CD44^+^CXCR5^+^) and non-T_FH_ (CD44^+^CXCR5^−^) cells in transferred SMARTA cells was 2.2- and 2.1-fold higher than that in host-derived CD4^+^ T cells, respectively (**Figure 1D**). Collectively, these results suggest that Themis is involved in the differentiation of CD4^+^ T cells, including T_FH_ cells, during the early stage of chronic viral infection.

### Themis promotes T_FH_ cell differentiation at the peak of chronic viral infection

To ascertain the effect of Themis in T_FH_ cell differentiation, we infected Themis T-cell conditional knockout mice (*Themis^fl/fl^; dLck* mice, hereafter cKO mice) and wild-type control mice (*Themis^fl/fl^*, hereafter WT mice) (37) with LCMV and examined their GC response. In these cKO mice, Themis was efficiently deleted in both CD8^+^ and CD4^+^ T cells, but a homeostasis defect was only reported in the CD8^+^ T cell compartment (37, 40). We confirmed this result (**Supplementary Figure S1A**) and further found that basal levels of CD4^+^ T cell activation were comparable in cKO and WT mice (**Supplementary Figure S1B**). Interestingly, in a competitive lymphopenia-induced homeostasis model (**Supplementary Figure S1C**), cKO CD4^+^ T cells exhibited a homeostatic proliferation defect at late but not early time points (**Supplementary Figures S1D, E**), but to a much lesser extent than that reported in CD8^+^ T cells (37, 40).

**Figure 2:**
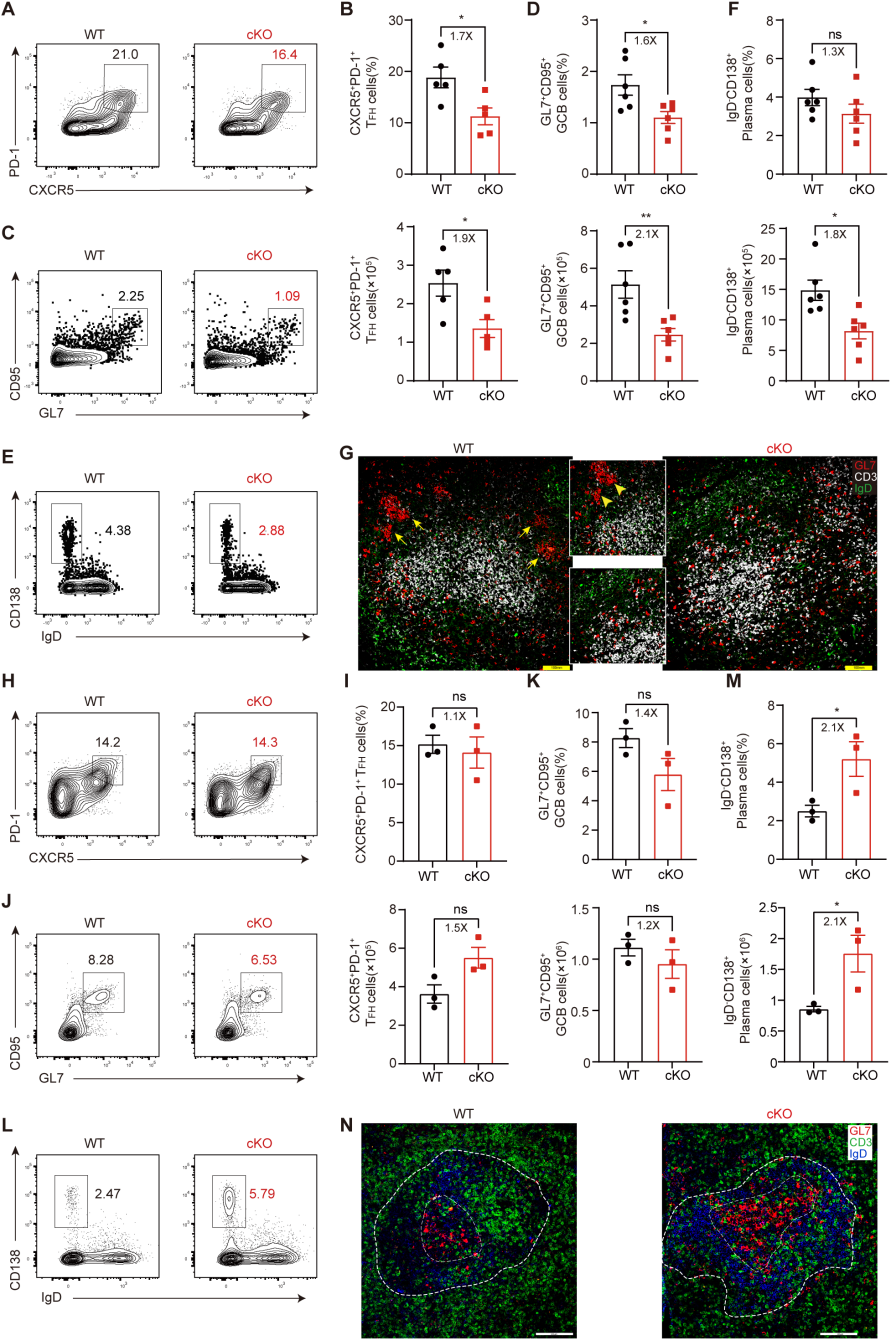
Themis regulates T_FH_ cell differentiation during the peak of chronic viral infection. **(A-G)** Mice were infected with LCMV C13 and analyzed at 8 dpi. **(A, B)** Analysis of GC-T_FH_ cells. Shown are representative FACS plots **(A)** and summary of the percentage and cell number **(B)**. **(C, D)** Analysis of GCB cells. Shown are representative FACS plots **(C)** and summary of the percentage and cell number **(D)**. **(E, F)** Analysis of plasma cells. Shown are representative FACS plots **(E)** and summary of the percentage and cell number **(F)**. **(G)** Representative immunofluorescence image of spleen sections from mice at 8 dpi, stained for GL7 (red), CD3 (white), and IgD (green). Adjacent zoom-in images highlight regions of interest. Scale bar: 100 μm. **(H-N)** Mice were infected with LCMV Arm and analyzed at 8 dpi. **(H, I)** Analysis of GC-T_FH_ cells. Shown are representative FACS plots **(H)** and summary of the percentage and cell number **(I)**. **(J, K)** Analysis of GCB cells. Shown are representative FACS plots **(J)** and summary of the percentage and cell number **(K)**. **(L, M)** Analysis of plasma cells. Shown are representative FACS plots **(L)** and summary of the percentage and cell number **(M)**. **(N)** Representative immunofluorescence image of spleen sections from mice at 8 dpi, stained for GL7 (red), CD3 (green), and IgD (blue). Adjacent zoom-in images highlight regions of interest. Scale bar: 100μm. Data are representative of at least two independent experiments (n=3–5 mice per group). In data summary plots, each symbol represents an individual mouse, the error bars represent the standard errors of the means (SEMs). P value was calculated by unpaired student’s t test, ns, not significant, *P < 0.05, **P < 0.01.

For LCMV C13 infection, at 8 dpi – the peak response – we found that the proportion and number of CXCR5^+^PD-1^+^ GC-T_FH_ cells in cKO mice was significantly reduced compared with those in WT mice (**Figures 2A, B**), and the generation of CD95^+^GL7^+^ GCB cells was also reduced (**Figures 2C, D**). Furthermore, although the proportion of CD138^+^IgD^–^ plasma cells was the same in WT and cKO mice, the number of these cells was decreased by 1.8-fold in cKO mice (**Figures 2E, F**). Consistent with the decrease in cell number in cKO mice, we found that the GC structure of cKO mice was very poorly delineated, with no clear shape, while the GC structure of WT mice was very clearly defined (**Figure 2G**).

In contrast to LCMV C13 infection, CD4^+^ T cells and antibodies are not required for viral control during acute LCMV infection, but GC responses still occur in this setting. We found that at 8 dpi, the generation of GC-T_FH_ cells was similar in WT and cKO mice (**Figures 2H, I**), as was the generation of GCB cells (**Figures 2J, K**). However, the number of plasma cells in cKO mice was significantly increased (**Figures 2L, M**), suggesting that GCB cell outflow may be enhanced in cKO mice. Accordingly, we found that the GC size of cKO mice was larger than that of WT mice (**Figure 2N**). Taken together, these results suggest that Themis deficiency has a more pronounced effect on T_FH_ cell differentiation and GC responses in chronic viral infection.

### Themis-deficient CD4^+^ T cells enhance humoral immunity and viral control in chronic LCMV infection

**Figure 3:**
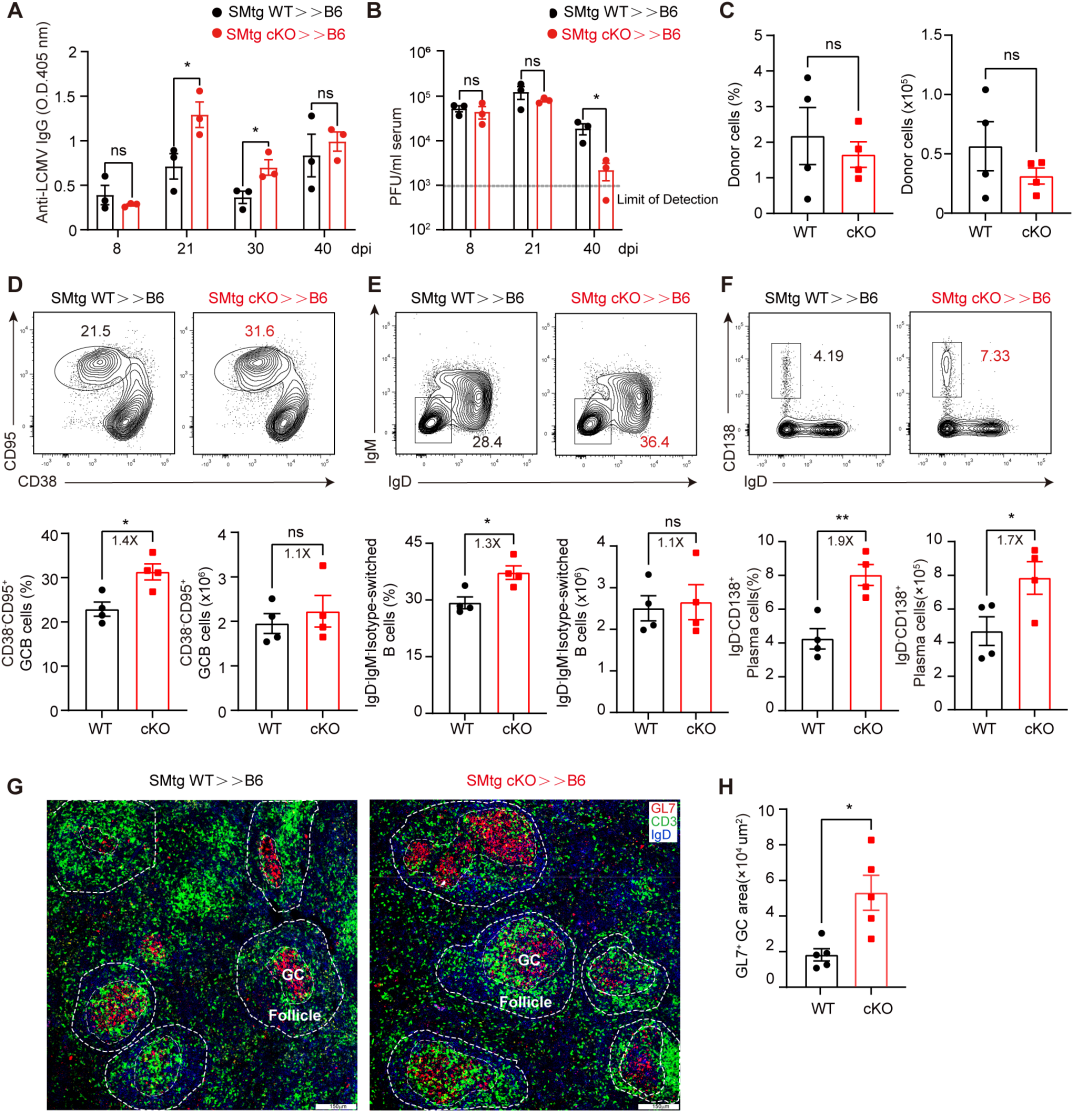
Themis-deficient CD4^+^ T cells augments antibody responses and facilitates viral clearance during LCMV C13 infection. Recipient mice were adoptively transferred with 1×10^4^ of naïve SMtg WT or SMtg cKO cells with different congenic markers and then infected with LCMV C13. **(A)** Serum abundance of anti-LCMV specific IgG were measured by ELISA at indicated timepoints. **(B)** Viral loads were determined by quantitative PCR at indicated timepoints. The dashed line indicates the limit of detection. **(C)** The frequency and number of SMtg WT and SMtg cKO donor cells at 21 dpi. **(D-F)** In-depth analysis of GCB cells **(D)**, isotype-switched B cells **(E)**, and plasma cells **(F)** at 21 dpi. Shown are representative FACS plots (top) and summary of the frequency and cell number (bottom). **(G)** Immunofluorescence image of the spleen section of recipients at 21 dpi, stained for GL7 (red), CD3 (green) and IgD (blue). Scale bar: 150 μm. **(H)** Summary of the area of GL7^+^ GC. Data are representative of at least two independent experiments (n=3–5 mice per group). In data summary plots, each symbol represents an individual mouse, the error bars represent the standard errors of the means (SEMs). P value was calculated by unpaired student’s t test, ns, not significant, *P < 0.05, **P < 0.01.

Although our above results suggest that Themis-deficient CD4^+^ T cells are defective in T_FH_ cell formation and in promoting GC responses at the peak of antiviral response, it is noteworthy that most cKO mice developed morbidity around 8 dpi, which may complicate our interpretation of the intrinsic role of Themis-deficient CD4^+^ T cells. To circumvent this, we generated SMARTA cells in cKO (SMtg cKO) and WT background (SMtg WT) and transferred equal numbers of both cell types into B6 recipient mice followed by LCMV C13 infection. Based on the aforementioned defects (**Figures 2A-G**), we expected that SMtg cKO cells might provide little help to B cells compared with SMtg WT cells. Contrary to that notion, we found that at 21 and 30 dpi, the abundance of anti-LCMV IgG antibody in mice receiving SMtg cKO cells were significantly higher than those in mice receiving SMtg WT cells (**Figure 3A**), indicating that the humoral immune response was enhanced in the mice receiving Themis-deficient SMtg T cells. Accordingly, we found that although the viral titers in the sera of mice receiving SMtg cKO or SMtg WT cells were comparable at 8 dpi and 21 dpi, the viral titers in mice receiving SMtg cKO cells were significantly reduced by 40 dpi compared with those in mice receiving SMtg WT cells, and the viral titers in some mice receiving SMtg cKO cells were even below the detection limit (**Figure 3B**).

The above findings prompted us to further analyze GC responses. For this, we chose 21 dpi because at this time point viral titers were comparable in both recipient mice, ruling out a potential effect of different viral load. Moreover, at this time point, the total number and percentage of both WT and cKO SMtg donor cells were roughly comparable (**Figure 3C**). Consistent with enhanced antibody production, we found that the frequencies of CD95^+^CD38^–^ GCB cells and IgD^–^IgM^–^ isotype-switched B cells were significantly increased in mice receiving SMtg cKO cells, although the absolute numbers of these cells were the same as those in mice receiving SMtg WT cells (**Figures 3D, E**). The number and percentage of CD138^+^IgD^–^ plasma cells were also markedly increased in mice receiving SMtg cKO cells (**Figure 3F**). Finally, immunohistochemical staining revealed that GL7^+^ GC were enlarged in the spleens of mice receiving SMtg cKO cells compared with those receiving SMtg WT cells (**Figures 3G, H**). Taken together, these results suggested the Themis-deficient LCMV-specific CD4^+^ T cells can enhance the humoral immune responses after transfer into recipient mice.

### Themis-deficient CD4^+^ T cells enhance the quantity of CD8^+^ T cells in chronic LCMV infection

**Figure 4:**
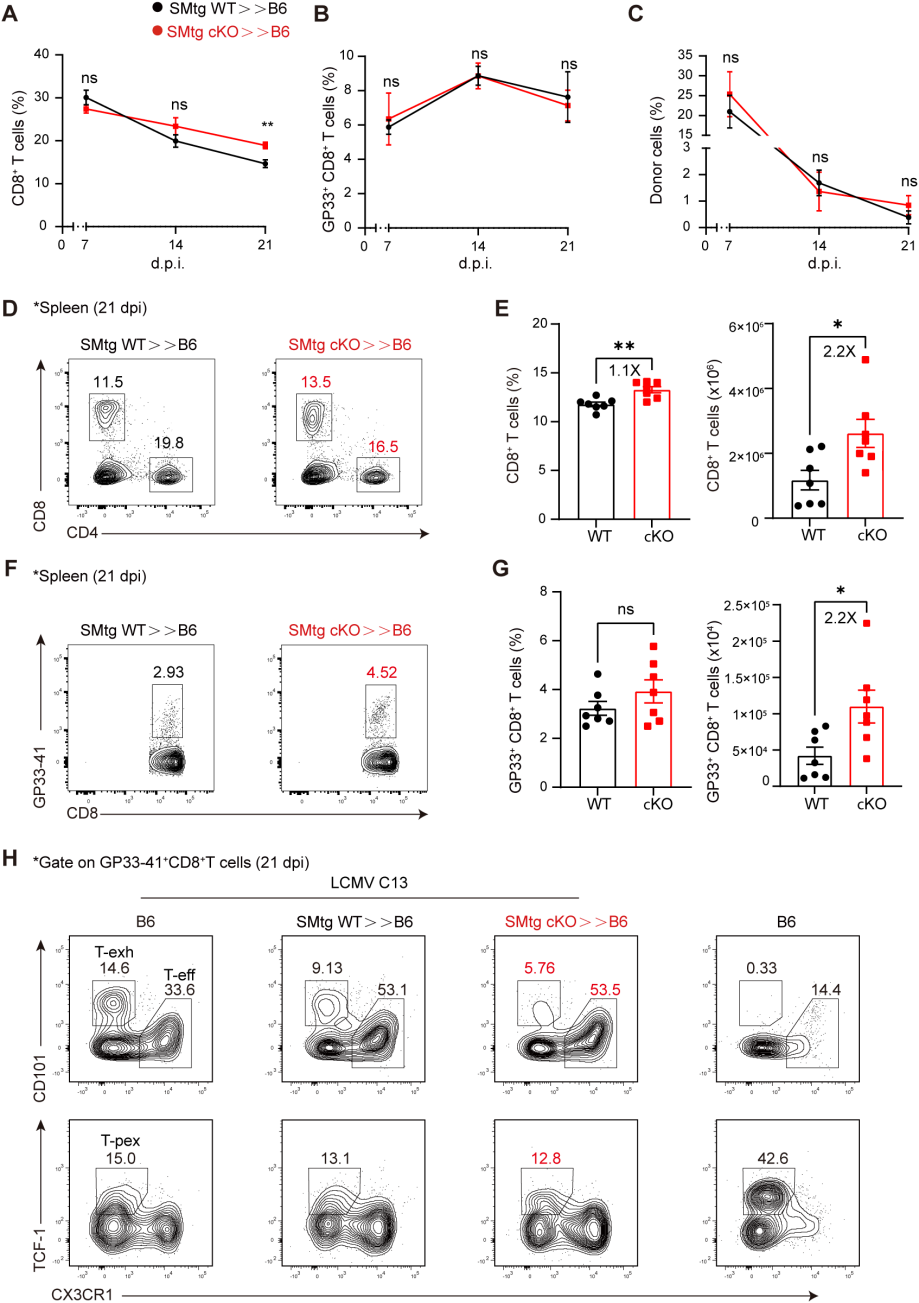
Themis-deficient CD4^+^ T cells enhances the quantity of CD8^+^ T cells in chronic LCMV Infection. C57BL/6J mice were adoptively transferred with 1×10^4^ naïve SMtg WT or SMtg cKO cells with different congenic markers and then infected with LCMV C13. A-C Peripheral blood were analyzed at indicated time points for total CD8^+^ T cells **(A)**, virus-specific GP33^+^CD8^+^ T cells **(B)**, and donor SMtg cells **(C)**. **(D, E)** Analysis of splenic CD8^+^ T cells from recipient mice at 21 dpi. Shown are representative FACS plots **(D)** and summary of the percentage and cell number **(E)**. **(F, G)** Analysis of GP33^+^CD8^+^ T cells gated on total CD8^+^ T cells from **(D)**. Shown are representative FACS plots **(F)** and summary of the percentage and cell number **(G)**. H Effector CD8^+^ T cells (CX3CR1^+^), terminally exhausted CD8^+^ T cells (CD101^+^), and T-pex cells (TCF-1^+^) were analyzed in LCMV C13 infected mice after gating on GP33^+^CD8^+^ T cells. Naïve B6 mice were included as a control for staining purpose. Data are representative of at least two independent experiments (n=3–5 mice per group). In data summary plots, each symbol represents an individual mouse, the error bars represent the standard errors of the means (SEMs). P value was calculated by unpaired student’s t test, ns, not significant, *P < 0.05, **P < 0.01.

Next, we wondered whether transferred SMtg cKO cells would enhance the function of endogenous CD8^+^ T cells, which normally undergo exhaustion and dysfunction during chronic LCMV infection. Again, we transferred equal amounts of naive SMtg cKO or SMtg WT cells into B6 mice, followed by LCMV C13 infection. We first examined the kinetics of endogenous total CD8^+^ T cells and LCMV-specific GP33–41 tetramer^+^ T cells in the blood. We found that, while the proportion of total CD8^+^ T cells was comparable in mice receiving SMtg WT and SMtg cKO cells at 7 and 14 dpi, it was slightly higher in mice receiving SMtg cKO cells at 21 dpi (**Figure 4A**). However, the proportion of LCMV-specific GP33–41 tetramer^+^ CD8^+^ T cells was the same at all time points (**Figure 4B**). Notably, we found that the proportions of SMtg cells from both donors were roughly equal and continued to decline synchronously during this period (**Figure 4C**). We next performed a more in-depth analysis at 21 dpi, as CD8^+^ T cells reach complete exhaustion at this stage. We found that while the proportion of total CD8^+^ T cells in the spleen of mice receiving SMtg cKO cells barely increased, their numbers doubled (**Figures 4D, E**). More importantly, the number of LCMV-specific GP33–41^+^ CD8^+^ T cells also doubled (**Figures 4F, G**).

In addition to cell number, we also examined whether the transferred SMtg cKO cells would influence the developmental status of exhausted CD8^+^ T cells. One model proposes that, during the early stage of infection, naive CD8^+^ T cells initially bifurcate into short-lived effector CD8^+^ T cells and stem cell-like T-pex cells. As infection persists, T-pex cells continue to self-renew, generating intermediate or transitionally exhausted CD8^+^ T cells and eventually differentiate into terminally differentiated exhausted CD8^+^ T cells at the late stage of infection (53–57). Consistent with this model, we found that at 21 dpi, effector CD8^+^ T cells (CX3CR1^+^), terminally exhausted CD8^+^ T cells (CD101^+^), and T-pex cells (TCF-1^+^) were clearly present in LCMV C13-infected mice (**Figure 4H**, far left). As expected, in naive B6 mice, effector cells were few in number and exhausted cells were absent, whereas TCF-1^+^ cells were predominantly naive cells (**Figure 4H**, far right). Furthermore, we observed a clear shift in CD8^+^ T cell subsets when SMtg cells were transferred. That is, the proportion of CX3CR1^+^ effector CD8^+^ T cells was significantly increased, while the proportion of terminally exhausted CD101^+^ CD8^+^ T cells was decreased (**Figure 4H**, top row), but no changes were found in TCF-1^+^ T-pex cells (**Figure 4H**, bottom row). However, no statistical difference was found between mice receiving SMtg cKO or SMtg WT cells (**Supplementary Figure S2A**). Taken together, these results suggest that, during chronic LCMV infection, transfer of Themis-deficient CD4^+^ cells boosts the total number of endogenous CD8^+^ T cells at the population level but does not affect their exhaustion status at the single-cell level. Nevertheless, the increase in the number of CD8^+^ T cells, especially LCMV-specific CD8^+^ T cells, together with the enhanced antibody response described above (**Figure 3A**), was sufficient to better control the virus (**Figure 3B**).

The benefits of Themis-deficient CD4^+^ T cells during chronic LCMV infection suggest that these cells may be used for cell therapy. However, the expansion of virus-specific CD4^+^ T cells has previously been shown to be associated with immunopathology, including cytokine storm, systemic inflammation, and death (58). To evaluate whether adoptively transferred Themis-deficient CD4^+^ T cells induce immunopathology, we analyzed various tissue sections from mice that received SMtg WT and SMtg cKO cells and found that at 8 dpi, the peak of the T cell response, no obvious damage was observed in the tissues examined (**Supplementary Figure S2B**). To formally test whether Themis-deficient virus-specific CD4^+^ T cells could be used to treat chronic LCMV infection, we injected these cells into previously infected mice and monitored viral load in the blood (**Supplementary Figure S2C**, top row). We found that mice transferred with SMtg cKO cells showed a trend toward benefit, as more mice had viral loads below the limit of detection at later time points compared with mice transferred with SMtg WT cells (**Supplementary Figure S2C**, bottom row). However, due to individual differences in mice, the statistical results were not significant.

### Themis promotes early stages of T_FH_ cell differentiation during chronic LCMV infection

**Figure 5:**
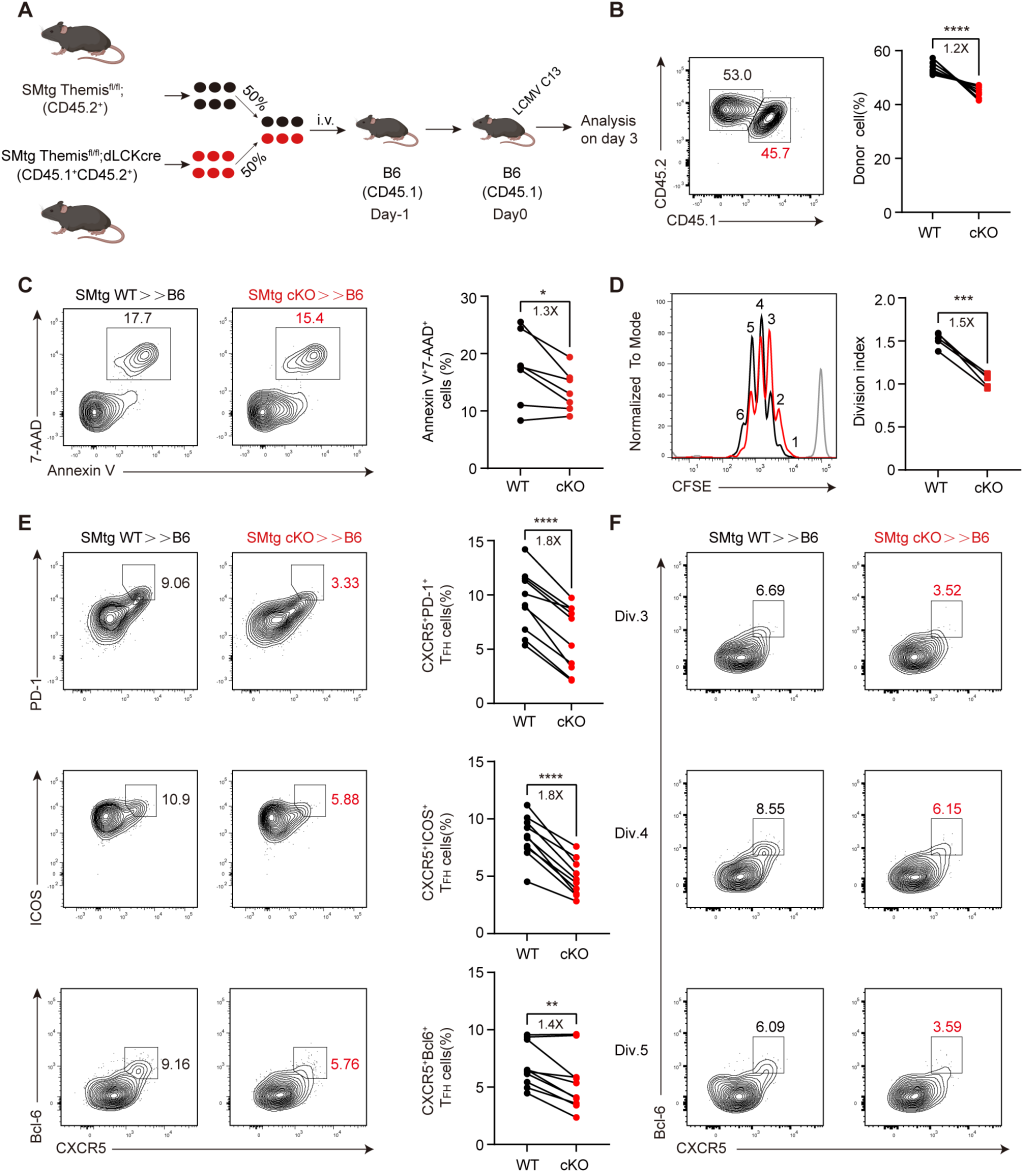
Themis promotes early T_FH_ cell differentiation in chronic infection. **(A)** Scheme of experimental setup. B6 mice were adoptively transferred with 2×10^6^ of equally mixed naïve SMtg WT and SMtg cKO cells, and then infected by LCMV C13. Mouse image obtained from BioRender. **(B)** The proportion of SMtg WT and SMtg cKO donor cells. Shown are representative FACS plots and summary of the percentage of cells. **(C)** Cell death analysis of transferred SMtg cells. Shown are representative FACS plots and summary of data. D-F. B6 recipient mice were adoptively transferred with CFSE-labeled naive SMtg WT or SMtg cKO cells and then infected with LCMV C13 and analyzed at 3 dpi. The proliferation of SMtg cells was determined by the CFSE profile. Shown are representative FACS plots and summary of the division index. Gray line in FACS plot indicates the CFSE profile of SMtg cells when transferred into naïve mice **(D)**. **(E)** FACS analysis of T_FH_ cells by indicated marker combination. Shown are representative FACS plots and summary of the data. **(F)** FACS analysis of T_FH_ cells in indicated cell division. Shown are representative FACS plots. Data are representative of at least two independent experiments (n=5–7 mice per group). In data summary plots, each symbol represents an individual mouse, the error bars represent the standard errors of the means (SEMs). P value was calculated by paired student’s t test, ns, not significant, *P < 0.05, **P < 0.01, ***P < 0.001, ****P < 0.0001.

T_FH_ cell differentiation is a multi-stage process, in which dendritic cells (DCs) are generally required for early T_FH_ cell differentiation (59), while B cells are required for further differentiation and completion of GC-T_FH_ cell maturation (60). Interestingly, our above results showed that, on the one hand, Themis deficiency impaired endogenous T_FH_ cell differentiation at the peak of chronic LCMV infection (**Figures 2A, B**), but on the other hand, transferred Themis-deficient CD4^+^ T cells enhanced GC responses and viral control at the late stage of infection (**Figures 3A, B**). These results suggested that Themis may play a stage-specific role in T_FH_ cell differentiation and exert opposing effects at different stages. To investigate this possibility and to exclude potential influences from environmental factors, we co-transferred equal numbers of naive SMtg WT and SMtg cKO cells with different congenic markers into B6 mice and then infected with LCMV C13 (**Figure 5A**). In addition, we advanced our analysis to 3 dpi for early T_FH_ cell differentiation, based on previous studies (50, 61) and our own results that Themis expression is upregulated in T_FH_ cells at this time point (**Figure 1D**). We first noticed a marked reduction in the proportion of SMtg cKO cells compared with SMtg WT cells (**Figure 5B**). To understand the reasons for this, we examined cell death in transferred SMtg cells. We found that the death rate of SMtg cKO cells was lower than that of SMtg WT cells as determined by Annexin V and 7-AAD staining (**Figure 5C**), ruling out cell death as a contributing factor to the reduction of SMtg cKO cells. Next, we examined their proliferation status by mixing equal numbers of naive SMtg WT and SMtg cKO cells (expressing different congenic markers), labeling them with CFSE, and then transferring them into B6 recipient mice. At 3 dpi, we found that SMtg WT cells exhibited more vigorous proliferation, while SMtg cKO cells displayed weaker proliferation. Specifically, CFSE profile revealed that SMtg cKO cells were predominantly in the third and fourth divisions, whereas WT cells had entered the fifth and sixth divisions (**Figure 5D**), suggesting that Themis deficiency impairs the proliferation of CD4^+^ T cells and is partially responsible for the reduction in cKO cell numbers.

We next analyzed the differentiation of T_FH_ cells and found that while T_FH_ lineages detected by CXCR5^+^ PD-1^+^, CXCR5^+^ ICOS^+^, or CXCR5^+^ Bcl-6^+^ all developed normally in SMtg WT cells, their proportions were significantly reduced in SMtg cKO cells (**Figure 5E**). In addition, we analyzed the expression of these markers and found that, except for Bcl-6, their expression in SMtg cKO cells was slightly decreased compared with those in SMtg WT cells (**Supplementary Figure S3A**). Previous studies have shown that CD4^+^ T cell differentiation choice occurs in the first few rounds of cell division, in which CXCR5 expression guides early T_FH_ cells to migrate to the border of B cell follicles and proceeds further T_FH_ cell differentiation (48, 49, 60). To investigate how early Themis begins to influence T_FH_ cell differentiation, we analyzed the relationship between T_FH_ cell differentiation and cell division by using the CFSE dilution profiles mentioned above. We found that the proportion of T_FH_ cells was reduced in SMtg cKO cells as early as division 3, and this trend continued until division 5 (**Figure 5F**; **Supplementary Figures S3B, C**). Taken together, these findings suggest that Themis promotes virus-specific CD4^+^ T cell expansion and differentiation into the T_FH_ cell lineage early in infection.

### Themis suppresses late stages of T_FH_ cell differentiation by preserving progenitor cells

**Figure 6:**
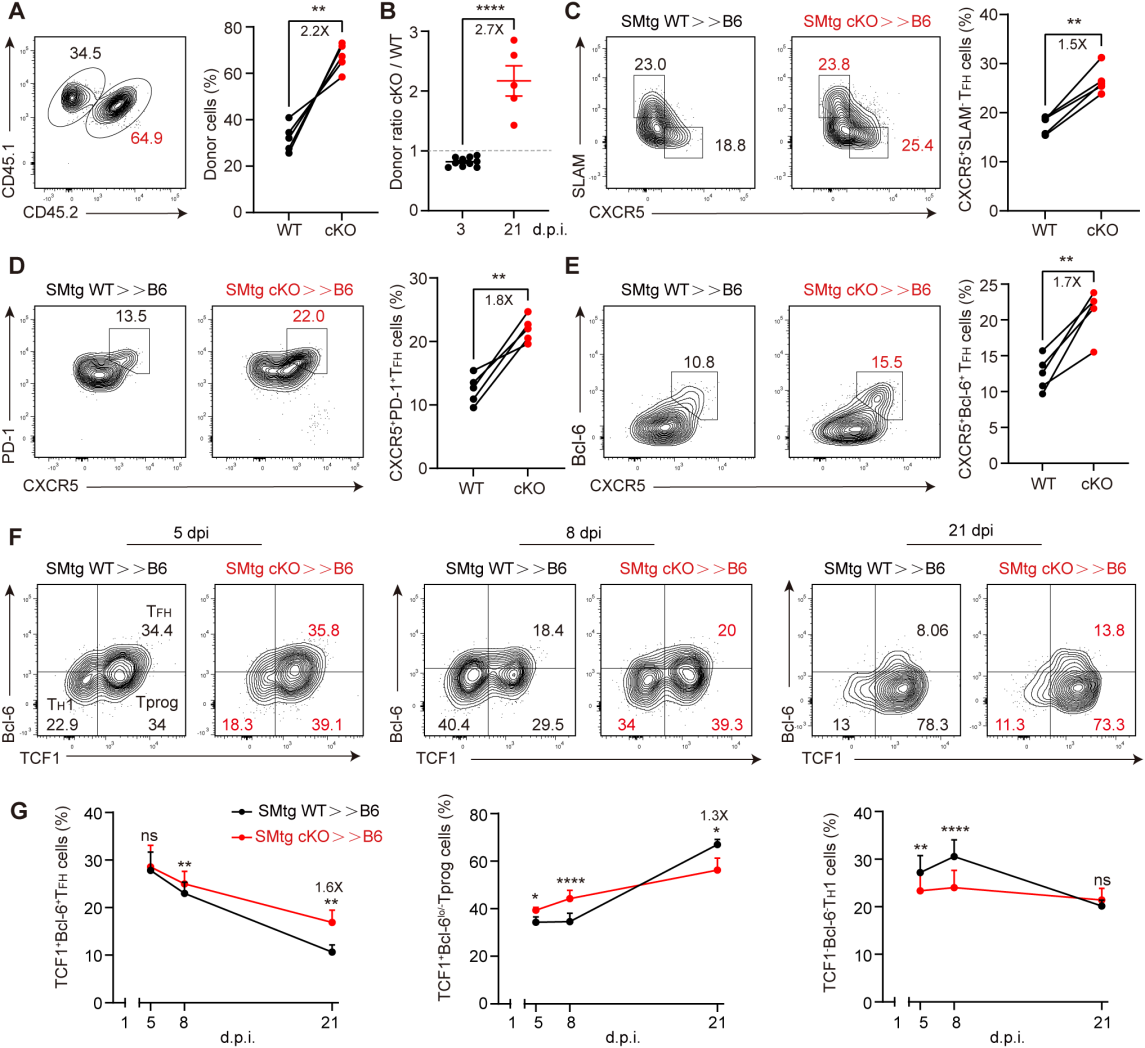
Themis inhibits late stage of T_FH_ cells differentiation. B6 mice were adoptively transferred with 1×10^4^ equally mixed naïve SMtg WT and cKO cells, and then infected by LCMV C13. **(A)** The proportion of SMtg WT and SMtg cKO donor cells at 21 dpi. Shown are representative FACS plots and summary of the percentage of cells. **(B)** Comparison of the ratio of SMtg cKO cells to SMtg WT cells at 3 dpi and 21 dpi. The dashed line indicates that the ratio of 1. C-E FACS analysis of CXCR5^+^SLAM^-^T_FH_ cells **(C)**, CXCR5^+^PD-1^+^ GC-T_FH_ cells **(D)**, and CXCR5^+^Bcl-6^+^ GC-T_FH_ cells **(E)**. Shown are representative FACS plots and summary of data. F, G Analysis of TCF1^+^Bcl-6^+^ T_FH_ cells, TCF1^+^Bcl-6^lo/–^ Tprog cells and TCF1^–^ Bcl-6^–^ T_H_1 cells at indicated time points. Shown are representative FACS plots and summary of data. Data are representative of at least two independent experiments (n=3–5 mice per group). In data summary plots, each symbol represents an individual mouse, the error bars represent the standard errors of the means (SEMs). P value was calculated by paired student’s t test **(A, C-E, G)** and unpaired student’s t test **(B)**, ns, not significant, *P < 0.05, **P < 0.01, ***P < 0.001, ****P < 0.0001.

Next, we investigated the effects of Themis on later stages of T_FH_ cell differentiation. The experimental setup was the same as before (**Figure 5A**), but analysis was performed at 21 dpi, as it was previously observed that transferred SMtg cKO cells enhanced GC response at this time point (**Figure 3**). Notably, this time we observed an increase in the proportion of SMtg cKO cells compared with that of SMtg WT cells (**Figure 6A**). This ratio of donor cells was completely opposite to what we observed at 3 dpi (**Figure 6B**), confirming that Themis indeed has a stage-dependent effect. Remarkably, we not only observed an overall shift in donor cell contribution, but also found a greatly increased proportion of T_FH_ cells in SMtg cKO cells, whether they were defined by CXCR5^+^SLAM^–^, CXCR5^+^PD-1^+^ or CXCR5^+^Bcl-6^+^ markers (**Figures 6C-E**). Recent studies have identified a population of TCF-1^+^Bcl-6^lo/–^ Tprog cells that emerge in the late stage of chronic viral infection that are capable of self-renewal and continuous generation of T_H_1 effector cells and T_FH_ cells (31, 32). Based on this model, we suspected that Themis might influence this newly-defined Tprog cell population during chronic LCMV infection. To test this, we examined the dynamics of this population in mice transferred with SMtg cells and subsequently infected with LCMV C13. Consistent with previous reports, we observed that the proportion of TCF-1^+^ Bcl-6^+^ T_FH_ cells in both SMtg cell types was continuously reduced, whereas the proportion of TCF-1^+^Bcl-6^lo/–^ Tprog cells was initially stable but then greatly increased. The proportion of TCF-1^–^ Bcl-6^–^ T_H_1 cells was transiently elevated and then decreased (**Figures 6F, G**). Although the dynamics of all the above cell subsets were comparable regardless of their genotype, there were quantitative differences. Specifically, at 21 dpi, the proportion of T_FH_ cells in SMtg cKO cells was 1.6-fold higher than that in SMtg WT cells, but the proportion of Tprog cells in SMtg cKO cells was 1.3fold lower than that in SMtg WT cells (**Figure 6G**). No difference was found for T_H_1 cells at this stage between two types of SMtg cells (**Figure 6G**). Collectively, these results suggest that Themis plays a crucial role in shaping the long-term differentiation of CD4^+^ T cells during chronic LCMV infection by preserving the Tprog cell pool. In the absence of Themis, CD4^+^ T cells readily differentiate into T_FH_ cells, while progenitor cells are depleted, leading to enhanced GC responses and antibody production, as well as better protection against persistent viral infection.

### ScRNA-seq reveals Themis inhibits CD4^+^ Tprog cell differentiation into T_FH_ via reprogramming of multiple transcription factors

**Figure 7:**
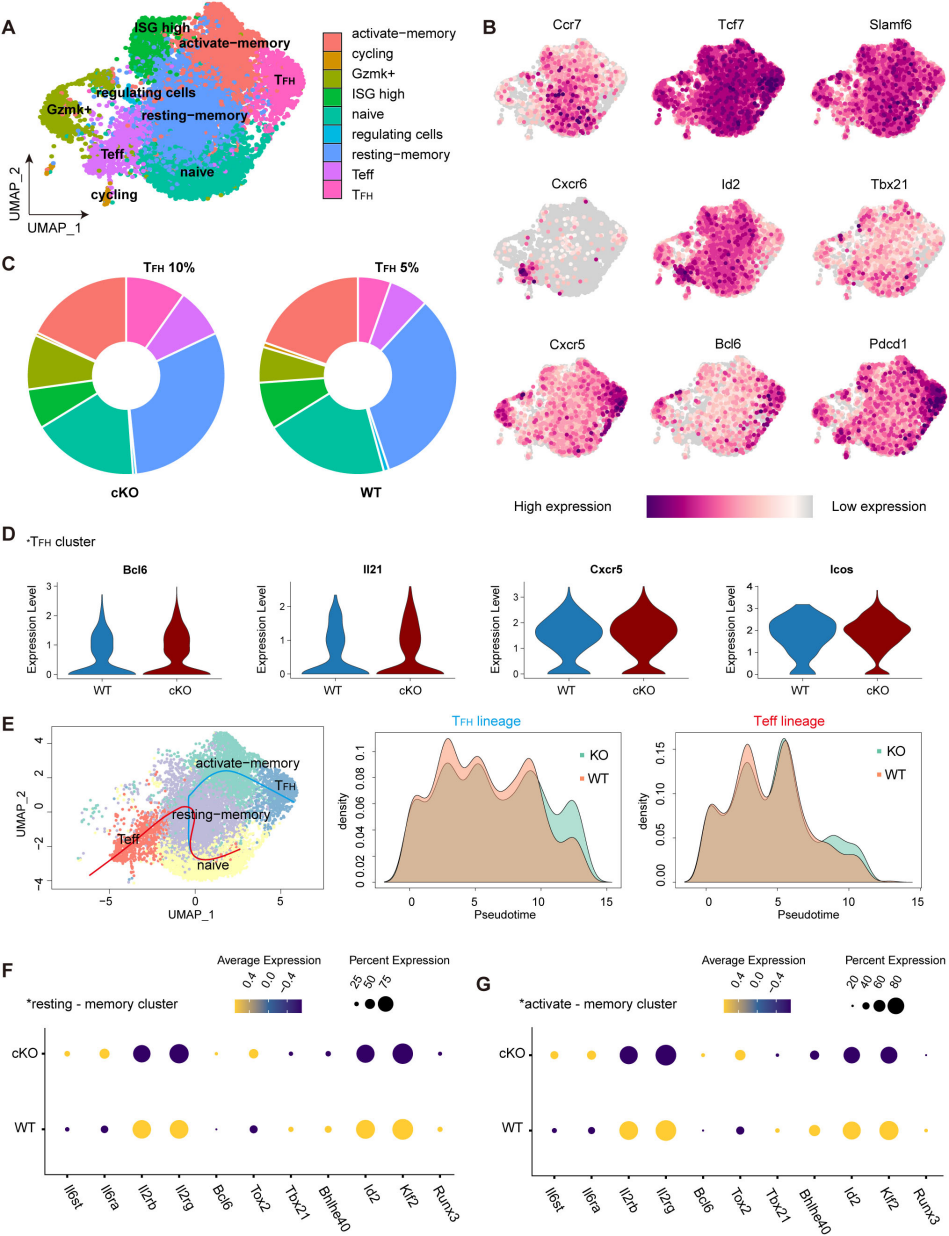
Single-cell RNA sequencing reveals the impact of THEMIS deficient on CD4^+^ T cell differentiation during chronic LCMV infection. **(A)** UMAP visualization of SMtg WT and cKO CD4^+^ T cells from LCMV C13 infected mice at 21 dpi. Cells were clustered based on scRNA-seq gene expression profiles and were color-coded by phenotypic clusters. **(B)** Feature plots highlighting the expression of key cluster-defining markers across CD4^+^ T cell populations. **(C)** Pie charts showing the proportion of cells from SMtg WT and cKO conditions within each phenotypic cluster. **(D)** Violin plots displaying the expression of key genes in SMtg WT and cKO CD4^+^ T cells within the T_FH_ cluster. **(E)** Pseudotime trajectory analysis of CD4^+^ T cells at 21 dpi, showing differentiation towards the T_FH_ and Teff lineages. Density plots depicted the distribution of SMtg WT and cKO cells along the pseudotime axis. T_FH_ lineage (middle panel); Teff lineage (right panel). F Dot plot illustrating the relative expression of differentially expressed genes between SMtg WT and cKO CD4^+^ T cells in the resting-memory cluster. G Dot plot showing the relative expression of differentially expressed genes between SMtg WT and cKO CD4^+^ T cells in the activated-memory cluster.

Our above results demonstrated that Themis inhibits the differentiation of newly defined TCF-1^+^Bcl-6^lo/–^ Tprog cells in the late stage of chronic LCMV infection. To obtain an unbiased overview of how Themis affects this process, we sorted the co-transferred SMtg WT and SMtg cKO cells at 21 dpi and performed single-cell RNA-seq (scRNA-seq) (**Supplementary Figure S4A**). Following quality control filtering (see Methods), a total of 11,400 single-cell profiles were obtained, comprising 6,318 cells and 5,082 cells from the SMtg cKO and SMtg WT compartments respectively. Dimensionality reduction and clustering based on gene expression profiles revealed 9 distinct cellular clusters (**Figure 7A**), which were classified and characterized using differentially expressed genes and markers. The distribution of some key gene expressions across these clusters is presented (**Figure 7B**, **Supplementary Figure S4B**). As expected, the naive cell cluster highly expressed *Ccr7*, *Tcf7*, *Slmaf6*. The effector T cell (Teff) subpopulation was identified by the unique expression of *Cxcr6*, *Id2*, *Tbx21*. The T_FH_ cluster predominantly expressed high levels of *Cxcr5*, *Bcl6*, *Pdcd1*. Other subsets were also captured and classified in accordance with previous reports (32) (**Supplementary Figure S4B**).

We found that the cellular distribution in SMtg WT cells at 21 dpi was consistent with previous reports (32), confirming our analysis approach. Remarkably, in the SMtg cKO cells, the proportion of T_FH_ cluster doubled, increasing from approximately 5% to 10% (**Figure 7C**), a result that aligns with our previous phenotypic analysis (**Figures 6C-E**). To further compare T_FH_-associated genes within the T_FH_ cluster between SMtg cKO and SMtg WT cells, we used violin plots to examine their expression. T_FH_ cluster from SMtg cKO group displayed significantly higher levels of *Bcl6*, *Il21*, *Cxcr5*, and *Icos* compared to SMtg WT (**Figure 7D**). We also analyzed Teff-related gene expression profiles within the Teff cluster, which showed distinct patterns between the two groups (**Supplementary Figure S4C**). Collectively, these results demonstrate that, during chronic LCMV infection, SMtg cKO cells exhibit an increased propensity to differentiate into T_FH_ commitment.

Trajectory inference with Slingshot 2 (see Methods) provided deeper insight into the developmental relationships between CD4^+^ T cell clusters, confirming that naïve CD4^+^ T cells transitioned through memory subsets before diverging into T_FH_ or Teff lineages. This is indeed consistent with previous studies showing that memory-like CD4^+^ T cells serve as progenitors that sustain Teff and T_FH_ cells during chronic LCMV infection (31, 32). However, a striking difference emerged in the density patterns between SMtg cKO and SMtg WT cells. That is, SMtg WT cells were more prevalent during the early stage of differentiation, whereas SMtg cKO cells exhibited accelerated progression toward terminal T_FH_ and Teff states (**Figure 7E**). The pseudotime analysis underscored the pivotal role of memory cells in the differentiation into T_FH_ and Teff lineages. Transcriptional regulators in resting-memory and activated-memory subsets from SMtg cKO and SMtg WT cells were further analyzed. Compared with SMtg WT cells, the expression of cytokine receptors *Il6ra* and *Il6st*, which are associated with T_FH_ cell differentiation, was increased in SMtg cKO memory subsets. In contrast, the expression of cytokine receptors *Il2rb* and *Il2rg*, which are more closely associated with Teff cell differentiation, was decreased in SMtg cKO memory subsets. In addition, transcription factors *Bcl6* and *Tox2*, associated with T_FH_ lineage potential, were upregulated in SMtg cKO memory subsets, while *Runx3*, *Klf2*, *Id2*, *Bhlhe40*, and *Tbx21*, linked to Teff differentiation, were downregulated (**Figures 7F, G**). These data suggest that Themis deficiency depletes the Tprog pool to promote the differentiation of CD4^+^ T cells into T_FH_ cells during chronic LCMV infection. To investigate the molecular basis of these trajectory differences, key transcription factors along pseudotime paths were analyzed. *Bcl6* and *Tox2*, key for T_FH_ cell differentiation, increased earlier and more prominently in SMtg cKO T_FH_ lineage, while remaining stable in SMtg WT. In contrast, Teff-associated factors *Klf2* and *Bhlhe40* were downregulated in SMtg cKO T_FH_ lineage. (**Supplementary Figure S4D**). These findings underscore a more pronounced and enhanced differentiation of CD4^+^ Tprog cells into T_FH_ cells in the absence of Themis.

## Discussion

The role of Themis in mature CD4^+^ T cells in the context of chronic viral infection has not been reported before. Our initial suspicion that Themis might modulate CD4^+^ T cell function during chronic LCMV C13 infection was based on our previous finding that CD4^+^ T cell depletion in infected cKO mice reduced their mortality to 60% (41). In contrast, without CD4^+^ T cell depletion, 80% of cKO mice died from CD8^+^ T cell-mediated lung injury. In this study, we report a novel role for Themis in regulating T_FH_ cell differentiation during chronic viral infection.

We found that in the early stage of chronic LCMV infection, the expression level of Themis was strongly positively correlated with the differentiation of T_FH_ cells in mice with endogenous polyclonal CD4^+^ T cells, suggesting that Themis is required for T_FH_ cell differentiation. However, this extreme T_FH_ cell bias was less pronounced in the transferred SMARTA cells, probably because in polyclonal mice, naive CD4 T^+^ cells are continuously supplied and activated, whereas in the transferred cells, these events occur in only a single wave. Our initial phenotypic analysis of T_FH_ cells was performed at 8 dpi, not only because our previous studies discovered the effect of CD4 T^+^ cell depletion at this time point (41), but also because we wanted to analyze the differentiation of T_FH_ cells in acute LCMV infection for comparison. Consistent with the dispensable role of CD4^+^ T cells in acute LCMV infection, Themis is not required for T_FH_ cell differentiation in this setting. In contrast, we confirmed that Themis is required for T_FH_ cell differentiation in chronic LCMV infection. However, we were concerned that the mortality and morbidity of cKO mice might extrinsically affect T_FH_ cell differentiation.

To eliminate the above concerns and exclude interference from other potential environmental factors, we performed SMtg cell co-transfer experiments and longitudinally monitored the differentiation of T_FH_ cells. It turns out that Themis in fact promotes T_FH_ cell differentiation early in infection but inhibits it later in infection. This unexpected inhibitory effect of Themis on T_FH_ cell differentiation at late stage of infection essentially explains our other finding that transfer of Themis-deficient CD4^+^ T cells helps control persistent viruses by enhancing GC responses and antibody production. This finding highlights the possibility that T_FH_ or CD4^+^ T cell function can be enhanced by targeted downregulation or degradation of Themis, which could be used for cell therapy of chronic viral infections. In fact, this has been attempted in CAR-T cells, where knockdown of Themis increased phosphorylation of CAR-CD3ζ, implying enhanced functionality of these CAR-T cells (62). However, this potential has not yet been formally tested in animal models. We took advantage of this study to conduct a preliminary test by transferring Themis-deficient SMARTA cells into mice infected with LCMV C13 and observed a beneficial trend, with more mice eliminating persistent virus compared to mice transferred with Themis-sufficient cells. Certainly, this aspect requires more rigorous and extensive *in vivo* evaluation and validation, not only in viral infection models but also in tumor models.

Our scRNA-seq data revealed that Themis preserves the Tprog cell pool and inhibits T_FH_ cell differentiation through a complex transcriptional network during chronic LCMV infection. This is indeed very similar to our findings in CD8^+^ T cells, where Themis preserves the progenitor cells T-pex and inhibits effector CD8^+^ T cell differentiation. These findings, together, highlight that during chronic viral infection and possibly in cancer, CD4^+^ and CD8^+^ T cells share a common strategy to maintain their long-term responses by reducing more potent, but also more harmful, effector cells. Thus, during an infection crisis, both types of T cells would be working together in the same direction, rather than working in separate directions.

Recent studies have also explored the role of Themis in CD4^+^ T cells from other aspects and experimental systems, such as autoimmune diseases. Using a CD4^+^ T cell transfer-mediated colitis model, a study demonstrated that Themis regulates CD4^+^ T cell effector function by controlling NFAT nuclear translocation and metabolic reprogramming (63). Although this study is instructive, it is important to note that the CD4^+^ T cells used were remnants of Themis germline knockout mice, which have severe innate defects in CD4^+^ T cell development. In another study, Themis has been shown to modulate the severity of experimental autoimmune encephalomyelitis (EAE) by regulating TCR-independent signaling and the production of pro-inflammatory cytokines in CD4^+^ T cells, mainly polarized T_H_1 cells (64). Further studies also suggested that Themis enhanced the activity of Vav1, a guanine nucleotide exchange factor that amplifies TCR signaling, thereby influencing the encephalitogenic potential of T cells (65). These T_H_1 cell-focused studies complement our studies on T_FH_ cells and they not only broaden our understanding of how Themis regulate CD4^+^ T cell function but also raise several challenges that require further in-depth investigation.

## Data Availability

The data presented in the study are deposited in the NCBI Gene Expression Omnibus (GEO) repository, accession number GSE284735.
