# Supplementary material for: Themis differentially regulates T follicular helper cell differentiation during early and late stages of chronic viral infection
Source: Front Immunol. 2025 Jul 24;16:1638178. doi: 10.3389/fimmu.2025.1638178 (PMC12328291; doi:10.3389/fimmu.2025.1638178)
Supplement: Supplementary file 1 [file DataSheet1.pdf]

Supplementary Material

For Themis differentially regulates T follicular helper cell differentiation during early and late stages of chronic viral Infection

Yuzhen Zhu[1†], Yuzhou Bao[1†], Ning Wang[1†], Qifeng Gan[2], Jian Tang[3], Yu Cong[2], Bowen Hou[1,6], Minxue Quan[1], Chaonan Yan[4], Siyi Liu[1], Shuo Lin[1], Xiaobin Zhang[2], Yanping Du[2], Lichao Hou[4], Nicholas R. J. Gascoigne[5,6], Bing Xu[1*], Guo Fu [1,3,7*] and Qifan Zheng[3*]

*** Correspondence:**
Corresponding Author
Emails: B. Xu (xubing@xmu.edu.cn), G. Fu (guofu@xmu.edu.cn), Q. Zheng (zhengqf@xmu.edu.cn)

The PDF file includes:

Fig. S1 to S4

Supplementary figure legends

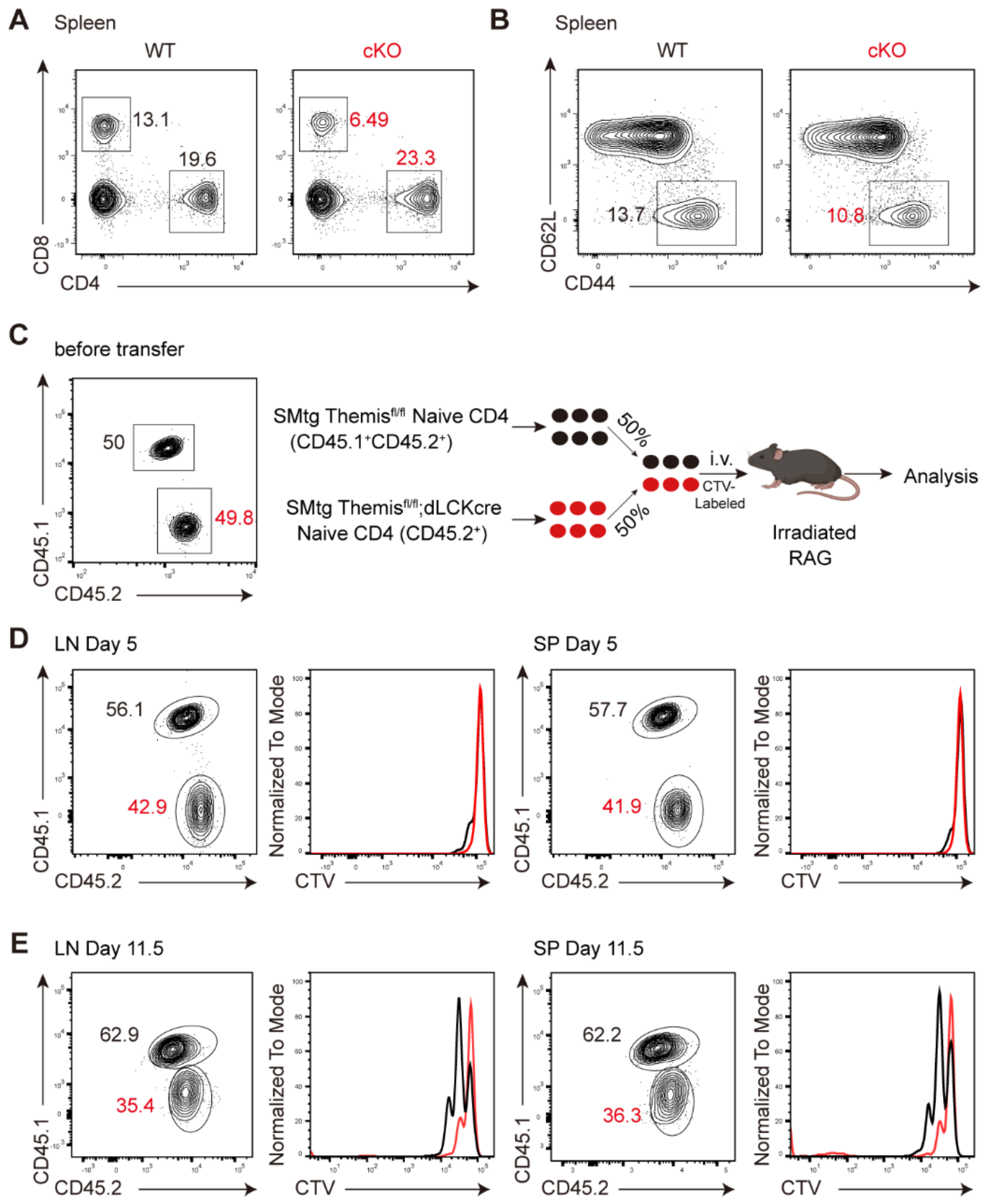

Fig. S1 Baseline analysis of CD4$^+$ T cells in cKO mice. Related to fig. 2. A Flow cytometric analysis of the proportion of CD4$^+$ T cells and CD8$^+$ T cells in the spleen of mice. **B** Flow analysis of basal level activation in CD4$^+$ T cells by CD44 and CD62L staining. **C** Experimental setup of competitive lymphopenia-induced homeostasis model. Purified SMtg WT and SMtg cKO cells with different congenic markers were mixed in equal amount (left), labeled with CTV, and then transferred into irradiated *Rag1*$^{-/-}$ knockout mice (right). **D, E** Flow analysis of the proportion and proliferation of transferred SMtg WT and SMtg cKO cells in lymph node (LN) and spleen (SP) at 5 days (D) or 11.5 days (E) post transfer. Data are representative of at least two independent experiments (n=3-5 mice per group).

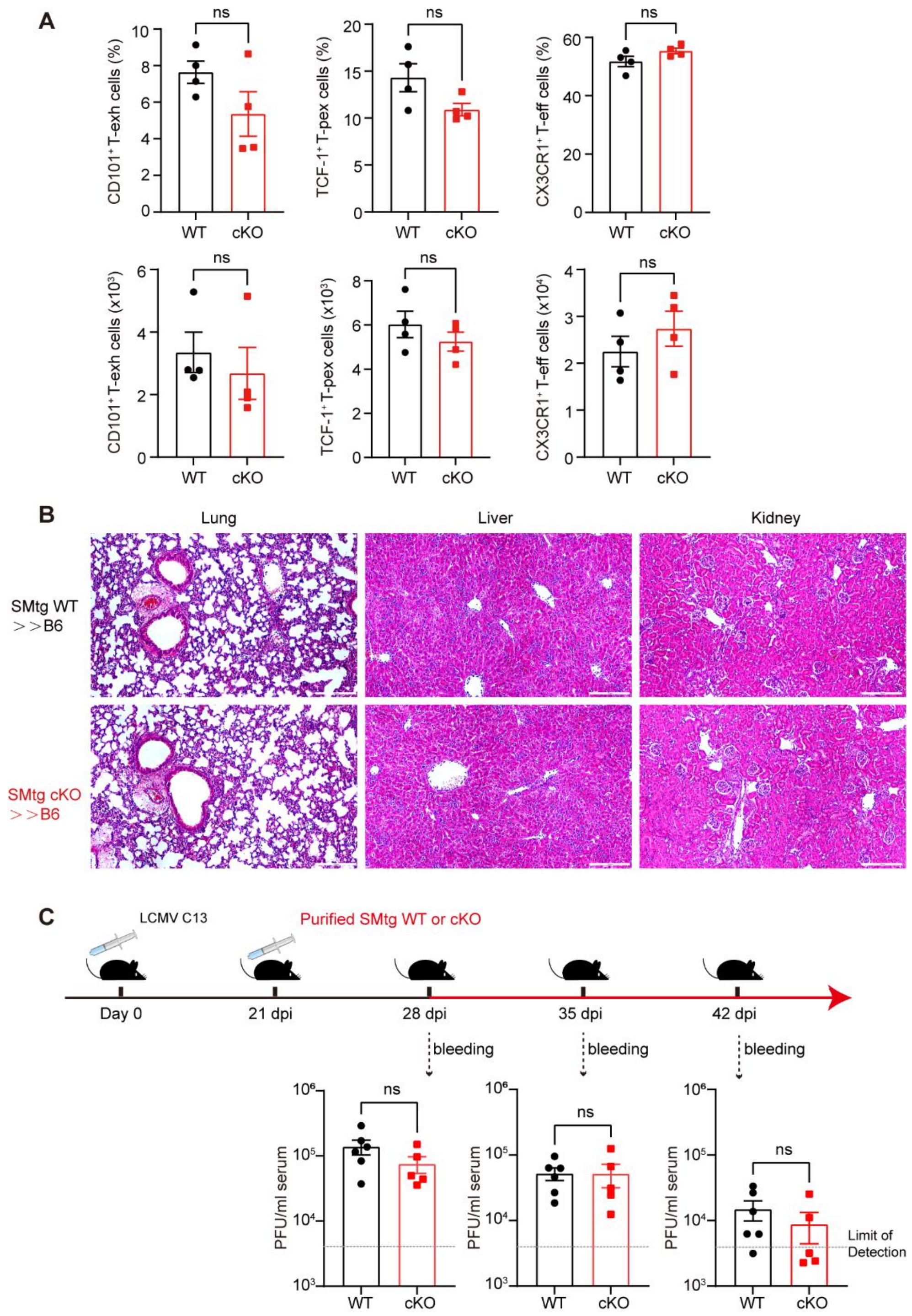

Fig. S2 Quantitation of endogenous CD8$^+$ T cell subsets in SMtg cell recipient mice at 21 dpi. Related to fig. 4. A Summary of the percentage and cell number of the indicated CD8$^+$ T cell subsets. **B** Mice were adoptively transferred with SMtg WT or SMtg cKO cells and then infected with LCMV C13. Tissue sections were prepared at 8 dpi, and analyzed by H&E staining. Scale bar, 200μm. **C** Mice were previously infected with LCMV C13 and transferred with SMtg WT or SMtg cKO cells at 21 dpi. The viral load in the blood was periodically monitored for 3 weeks. In data summary plots, each

symbol represents an individual mouse, the error bars represent the standard errors of the means (SEMs). P value was calculated by unpaired student's t test; ns not significant.

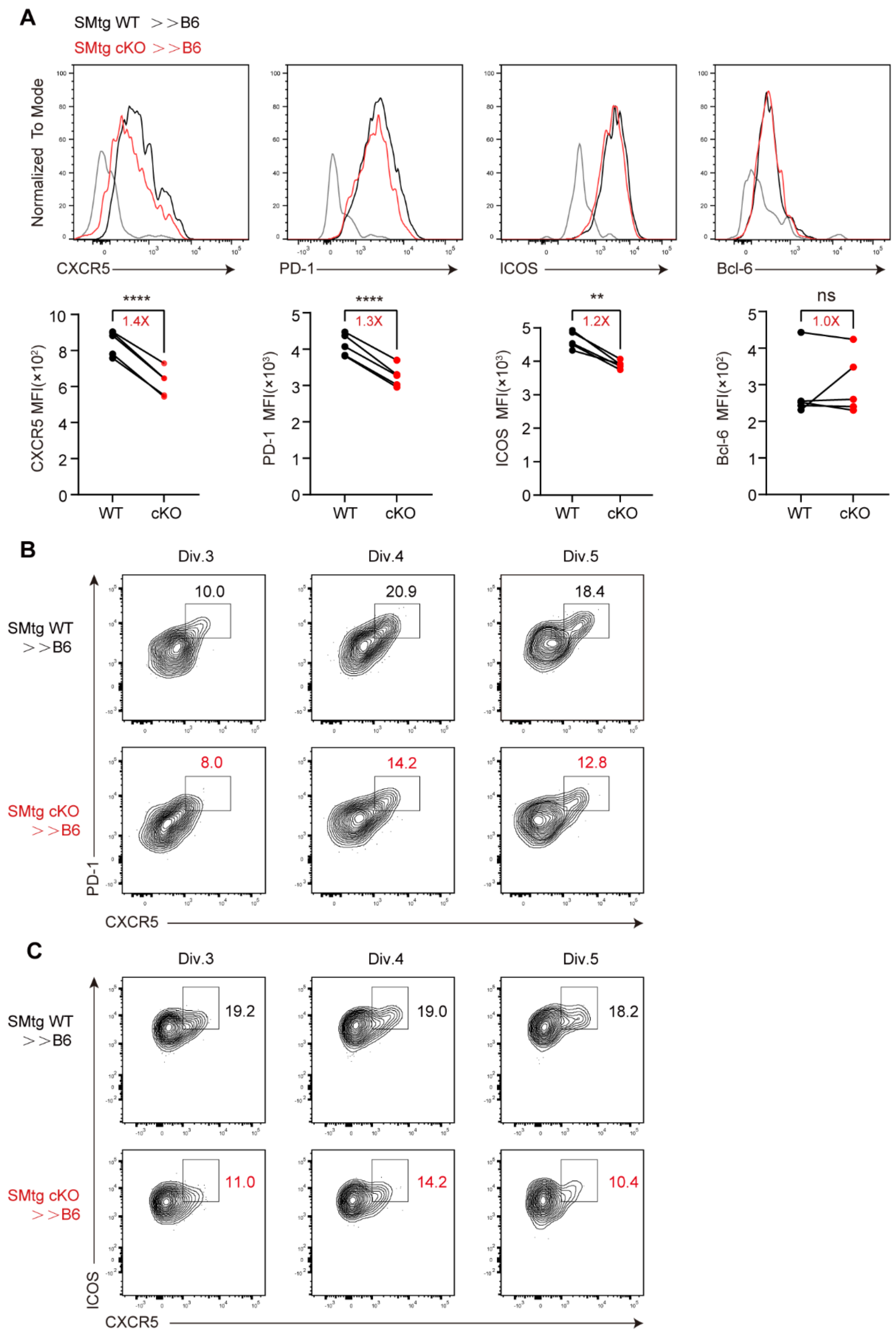

Fig. S3 SMtg cells co-transfer analysis at 3 dpi. Related to fig. 5. A The expression of indicated T_{FH}-related markers in transferred SMtg cells. Shown are representative flow cytometry histogram overlays (top panel) and summary of the MFI of indicated markers (bottom panel). The gray line in histograms represents naive control. **B, C** Flow analysis of $CXCR5^{+}PD\text{-}1^{+}$ T_{FH} cells (B) and $CXCR5^{+}ICOS^{+}$ T_{FH} cells (C) in indicated cell division. Shown are representative flow plots. Data are representative of at least two experiments (n=5-7 mice per group). In data summary plots, each symbol

represents an individual mouse, the error bars represent the standard errors of the means (SEMs). P value was calculated by paired student's t test; ns not significant, **$P < 0.01$, ****$P < 0.0001$.

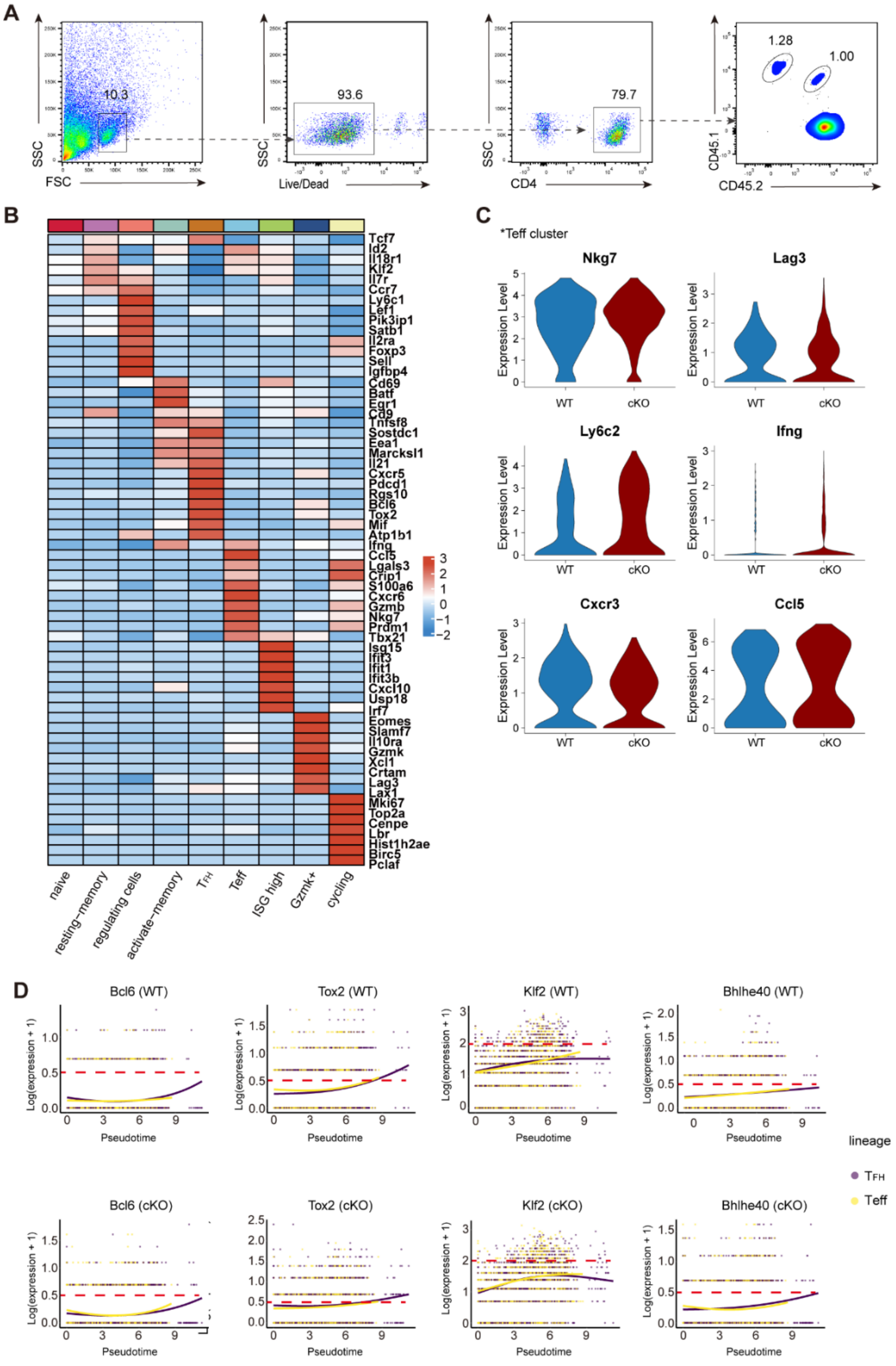

Fig. S4 scRNA-seq reveals heterogeneity within SMtg cKO and SMtg WT CD4$^+$ T cells in chronic LCMV infections. Related to fig. 7. A Sorting strategy of SMtg cKO and SMtg WT CD4$^+$ scRNA samples in chronic LCMV infections. **B** Heatmap of the differentially expressed markers for each scRNA-seq cluster. Colors represent normalized (z-scored) expression per gene among each cluster. **C** Violin plots displaying the expression of key genes in SMtg WT and cKO CD4$^+$ T cells within the

Teff cluster. **D** Gene expression dynamics of T_{FH} and Teff lineage markers along the pseudotime trajectory. SMtg WT gene expression patterns (top panel), SMtg KO gene expression trends (bottom panel).
